# Plant-based analogues to meat and dairy for sustainable food systems

**DOI:** 10.1017/S0029665126102237

**Published:** 2026-02-16

**Authors:** Sarah Nájera Espinosa, Genevieve Hadida, Anouk Reuzé, Leona Lindberg, Rosemary Green, Pauline Scheelbeek

**Affiliations:** 1 Department of Population Health, London School of Hygiene and Tropical Medicine, London, United Kingdom; 2 Centre on Climate Change and Planetary Health, London School of Hygiene and Tropical Medicine, London, United Kingdom; 3 School of Biological Sciences, Queen’s University Belfast, United Kingdom

**Keywords:** Climate change, meat reduction, dairy reduction, plant-based substitutes, sustainable diets, plant-based analogues

## Abstract

Various strategies across food systems are needed for a systemic change, with dietary shifts representing a meaningful pathway—particularly in high-income nations. Plant-based analogues (PBAs) that mimic animal-based foods, represent a promising strategy to facilitate such shifts because they require minimal behaviour adjustments. This review aims to synthesise nutritional, health and environmental evidence on PBAs by examining their benefits, challenges, and research gaps to inform and support evidence-based policy and practice. PBAs generally have lower greenhouse gas emissions, land use and water use than their animal-based counterparts. Nutritionally, PBAs are complex, varying across product brands, product types, processing techniques and primary ingredients. The limited health evidence shows that consumption of plant-based meat analogues tends to be associated with positive health outcomes, while consumption of some plant-based drinks can be linked to micronutrient deficiencies. Fortified PBAs can contribute to daily recommended intakes and sometimes provide more micronutrients than their animal-based counterparts, while also providing more fibre, and less energy and saturated fat. Despite these potential benefits, debates persist around processing classifications and their health implications. Given this complex landscape, assessing what kind of role PBAs could play in our food systems will demand product-specific evaluation, targeted dietary recommendations, and expanding the range of healthier PBAs. To advance the field and accelerate dietary shifts without unintended consequences, critical considerations include strengthening the nutritional evidence-base, classifying PBAs further for dietary recommendations and informed regulatory approaches, understanding processing effects and use of additives, and standardising environmental outcomes and research beyond single ingredients.

## Introduction

Structural transformation of the food system is essential for climate change mitigation and adaptation, and for improving food system resilience and population health. Despite a growing body of evidence indicating that plant-rich diets provide health and environmental benefits (1, 2), dietary patterns have remained largely unchanged, especially in high-income countries. Globally, around 40% of people do not adhere to recommended national dietary guidelines (3). In most countries, intakes of plant-based foods (e.g. whole grains, fruits, vegetables and legumes) remain below recommendations, while consumption of livestock products such as red and processed meat exceeds recommendations (3, 4).

Dietary choices are shaped by a complex interplay of factors. Culinary traditions play a key role in various cultures and often present substantial challenges for those pioneering and contemplating dietary shifts (5), especially for individuals trying to substitute traditionally meat-centric meals—an experience that varies widely depending on individual circumstances. Some successfully adopt plant-based diets (e.g. vegan and vegetarians) but encounter social barriers (6, 7), while other individuals struggle with the practical aspects of making these dietary changes effectively. The latter is associated with limited knowledge about nutritional content, health benefits, time, cost, or the cooking skills required to prepare healthy and sustainable meals using legumes, nuts and seeds, tofu, tempeh or seitan (8–11).

Despite these barriers, some successful population-level dietary shifts have been highlighted in the literature. For example, in the United Kingdom (UK), red and processed meat consumption has decreased by approximately 17%, and the proportion of self-identified vegetarians or vegans has risen by 3% over the past decade (12). However, these success stories only represent a small proportion of the population. Furthermore, evidence from UK purchasing supermarket data, has shown that pathways towards meat reduction are not always associated with healthier and sustainable substitutions. Carr et al. (13) identified two consumer clusters, one that shifted towards healthier and sustainable options, while the other cluster had variable substitutions among households, but they generally replaced meat with less healthy snacks such as snacks, prepared foods, biscuits and cakes.

**Figure 1:**
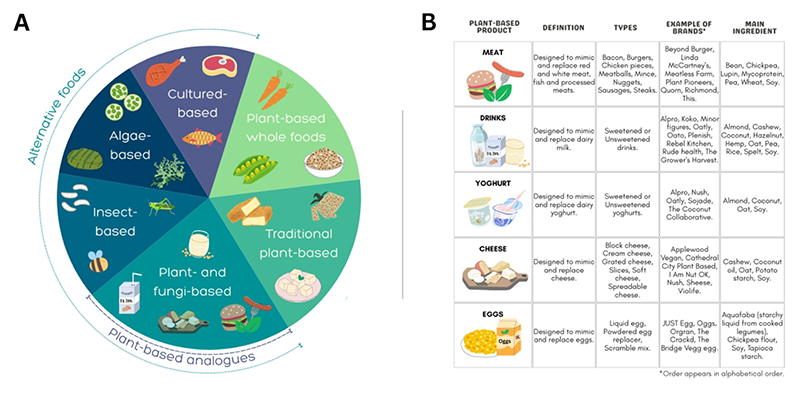
Types of alternative foods designed to mimic animal products and specific categories of plant-based analogues. Pane A shows all types of alternative foods including novel and plant-based whole foods. Pane B shows the definition, type, example of product brands and main ingredient for each category of plant-based analogues. 677×381mm (72 × 72 DPI)

Given the growing need for food systems transformation, the novel alternative foods landscape (e.g. cultured or cell-based, algae-based, insects, and fungi- or plant-based analogues) has emerged in recent years promising to support dietary transitions (See Figure 1, pane A). The growing interest in healthy and sustainable diets is thought to have driven increases in supply, demand, and investment, particularly in the plant-based analogue (PBA) sector (14–16). PBAs are fungal and plant-derived products that mimic the appearance and functionality of commonly consumed animal-based foods such as meat (processed and lean cuts) and dairy such as plant-based meat (e.g. mince, sausages, burgers), plant-based dairy (e.g. drinks, yoghurt, cheese) and plant-based eggs (See Figure 1, pane B). Among available novel alternatives, PBAs are already marketed and generally accepted by consumers due to their versatility and familiar ingredients, including fungi (i.e. mycoprotein) or plants (e.g. legumes, cereals) (17).

Alongside this increased supply and demand of PBAs, the academic literature on PBAs has also increased rapidly. There are numerous systematic reviews examining different aspects of PBAs, including their nutrient composition (18–21), production and processing (22, 23), food safety (24, 25), market predictions (26), and health (27, 28). Other reviews have assessed health and environmental outcomes for a specific category of PBAs, such as focusing on plant-based drinks (29) or plant-based meats (27, 30–32). Only one review assessed the nutritional, health and environmental outcomes across multiple PBA categories (33).

A number of these reviews have highlighted that PBAs could facilitate dietary changes, especially in high-income countries, because they require minimal dietary behaviour change if used to replace animal-based foods, especially processed meats. Current evidence also indicates PBA consumption occurs alongside animal-based food consumption (34). However, uncertainty and confusion remain regarding their nutritional content, nutrient bioavailability and bioaccessibility, environmental impacts and the degree of processing. This review aims to synthesise and discuss the current evidence on PBAs by examining the health, nutritional and environmental benefits, challenges, and research gaps to inform public health and environmental strategies and support evidence-based policy decision-making in high-income countries.

## Determinants of health, nutritional and environmental outcomes in plant-based analogues

Current research on PBAs is complex, with emerging evidence, several knowledge gaps, and promising but limited findings. The evidence shows that the primary ingredients (e.g. mycoprotein, legume), type of product (e.g. drink, meat), processing techniques, and product brand of PBAs (e.g. Quorn, Linda McCartney, Oatly etc) determine nutritional, health and environmental outcomes of PBAs (33). In the next section we have discussed in detail current evidence on health, nutritional and environmental outcomes for PBAs. See Table 1 for a summary of the key findings for each outcome.

### Human health outcomes

In this section, we have summarised the evidence across all PBAs (meat, drinks and other products) in relation to human health outcomes.

Short-term health studies (<1 day up to 56 days) suggest potential health benefits, particularly for plant-based meats; however, the field faces critical limitations in scope, duration, and population diversity that must be addressed to inform evidence-based dietary recommendations. Based on a comprehensive review published in 2024 (33), plant-based meat studies have mainly focused on mycoprotein, soy or pea protein products. Plant-based drink research primarily covers almond- and soy-based options (< 1day up to 30 days). Most studies have mainly focused on healthy adults (35–43).

While small, short-term trials provide valuable insights, long-term randomised controlled trials and prospective cohort studies are needed to more accurately evaluate the sustained health effects of PBAs consumption. This is particularly important because short-term outcomes are often confounded by individual variability in diet, health status, and consistency of consumption. Longer trials can help minimise these variables and provide a more accurate understanding of the sustained effects of PBAs.

A recent trial (44) illustrated these complexities by examining metabolic effects (including natural food matrices and their impact on satiety and glycaemic regulation) of various plant-based dairy products (sweetened plant-based drinks, yoghurt and cheese). The study found that while these PBAs were more effective in reducing post-meal appetite than their animal-based counterparts, they were less effective at lowering post-meal blood glucose levels. Based on these findings, the authors suggested that certain plant-based dairy products might compromise nutritional intake and glycaemic management (44). However, since this intervention used products with 15-25 additives and sweetened plant-based drinks, these results cannot be extrapolated to other types of PBAs such as those that are unsweetened and contain fewer additives.

The following sub-sections present the health evidence for plant-based meats and drinks in more detail. However, it is important to note that, across all PBA categories, data on the long-term health outcomes are lacking. Additional evidence is still required for plant-based drinks other than soy and almond-based, and other PBA categories such as plant-based yoghurt, cheese or egg analogues. Such studies are essential for developing tailored guidelines that mitigate potential risks for all individuals, including vulnerable populations (e.g. elderly, children, individuals with underlying diseases).

#### Evidence on plant-based meats

This sub-section presents the evidence for plant-based meats in relation to health outcomes. Based on a comprehensive review published in 2024 (33), to date, plant-based meat studies have mainly focused on health outcomes associated with their consumption, typically in healthy and overweight adults, over study periods ranging from less than a day to eight weeks, by substituting meat products with plant-based meats of equivalent caloric value.

Regarding mental health outcomes, the first randomised dietary intervention trial in healthy adults found no significant difference in psychological outcomes between groups consuming weekly amounts of red meat versus PBAs alongside a balanced vegetarian diet (45).

With respect to physical health, in our previous systematic review, Nájera Espinosa et al. (33) reported that mycoprotein-based meat consumption, instead of white and red meat, had beneficial effects associated with lower glycaemic markers, reduced energy intake, increased fibre intake, decreased insulin release, and positive microbiome changes. Similarly, legume-based meat consumption reduced cardiovascular risk markers, including trimethylamine-N-oxide levels and low-density lipoprotein cholesterol, while supporting beneficial gut bacteria growth. Three meta-analyses confirmed these findings (27, 28, 46), showing that substituting meat with legume-based and mycoprotein-based meats may lead to reductions in total cholesterol, low-density lipoprotein, body weight and triglycerides. Another review found positive cardiovascular outcomes of consuming plant-based meats, despite concerns about processing and higher levels of sodium (47).

While results from recent reviews are encouraging, some recent studies have reported neutral findings associated to plant-based meat consumption. A secondary analysis did not observe any differences in selected biomarkers of inflammation, when comparing legume-based meats with red meat of equivalent caloric value (43). Furthermore, in a randomised control trial in healthy Asian participants, Toh et al. (48) found that individuals did not exhibit any positive or negative cardiometabolic effects associated with the consumption of plant-based meats.

The short-term health findings associated with increasing PBA intake point to a promising direction for dietary adjustments, particularly among people advised to lower processed meat consumption for health reasons. This offers a great opportunity for individuals to begin shifting their diets, without sacrificing their sensory enjoyment. Processed meats are classified as “carcinogenic to human” (group 1) by The International Agency for Research on Cancer for their negative health outcomes and high content of saturated fat, sodium, energy density and preservatives (49, 50). For instance, in the UK, despite decreased intake of processed meat over the past decade (from 33.8 to 26.8 grams per day) (12), average intakes still account for almost half of the total national recommended intake for red and processed meat (51). Therefore, additional dietary changes are needed to further reduce processed meat intakes.

#### Evidence on plant-based drinks

This sub-section presents the evidence for plant-based drinks in relation to health outcomes. Evidence on plant-based drinks remains limited, with no published prospective cohort studies and research that has focused only on healthy adults (52–54).

Our systematic review, Nájera Espinosa et al. (33), identified micronutrient deficiency concerns in soy- and almond-based drinks, which have been linked with lower iodine intake and tooth demineralisation. The same review found that soy-based drinks produced similar glycaemic responses to dairy milk when consumed with white bread, though this was through different biological pathways (33).

Despite these micronutrient challenges, a recent systematic review and meta-analysis suggest that substituting cow’s milk with soy drinks (sweetened or unsweetened) is not associated with increased cardiometabolic risk factors (55). Moreover, this substitution showed certain benefits such as improved blood lipid profiles and lower blood pressure, without negative outcomes in other cardiometabolic markers.

Regarding oral health, Shkembi et al. (56) reviewed dental health impacts and concluded that plant-based drinks are more cariogenic than bovine milk due to added sugars, higher acidity, lower buffering capacity, and reduced calcium bioavailability, despite similar labelled content. However, most studies in this review were in vitro studies (there was only one randomised control trial) and focused on sweetened and unsweetened soy- and almond- based drinks.

Current evidence present a mixed picture of benefits and concerns that warrant careful consideration. Comprehensive research evaluating other plant-based drinks made from a wider range of ingredients, and their effects on both oral and overall health outcomes, remains urgently needed to inform evidence-based dietary recommendations.

### Nutritional composition

In the following section, the evidence on plant-based meats and plant-based dairy is considered in relation to nutritional composition. Several studies have assessed the nutritional composition of various PBAs, including meat, drinks, yoghurt, and cheese analogues (33). Similar to health outcome research, evidence regarding plant-based cheese and yogurt analogues remains limited.

However, the existing research demonstrates considerable diversity in primary ingredients across PBA categories. Plant-based drinks extend beyond soy and almond-based products to include numerous other types (e.g. oat and coconut), while plant-based meat analogues encompass legume-based, cereal-based, and mycoprotein-based options. This diversity contributes to a variability in nutritional profiles within PBA categories.

Despite this growing body of research, a significant methodological limitation persists; much of the available nutritional data is derived from web-scraping or product labelling rather than direct analytical samples, highlighting a critical need for more rigorous laboratory-based nutritional composition analysis.

At the macronutrient level (energy density, saturated fat, fibre, total sugar), there is considerable variability across PBAs categories (e.g. meat or dairy); however, median values across each primary ingredient and PBA type indicate that PBAs generally present better nutritional profiles relative to their animal-based equivalents, particularly to processed meats (33, 57). This nutritional advantage is particularly relevant because diets high intakes of saturated fat and energy-dense foods, combined with low fibre intake, are associated with the highest dietary burden of disease (58). Replacing animal-based products with PBA could help to mitigate these risks, particularly when substituting processed meats.

These nutritional profiles could provide substantial health benefits in high-income countries, where consumption deviates substantially from recommended levels. Similarly, certain PBAs, like plant-based meats and drinks, contain portions of vegetables, legumes and nuts (33), which could help address the inadequate intake of these food groups commonly observed in high-income countries, particularly among lower-income households (59). There is also substantial protein content variability across PBAs, but not all PBA median protein values match their animal-based protein content [see supplementary data in (33)]. Among all PBA categories examined, mycoprotein-based and legume-based products (including both meat and drinks analogues) show nutritional characteristics suitable for a healthy diet.

Nevertheless, most PBAs in this study contained sugar (33). While the total sugar content in plant-based meats is higher than their animal-based counterparts, other PBA categories (e.g. plant-based drinks and yogurts) show greater variation and some even contain less sugar than their animal-based counterparts. Although the total sugar content in most PBA categories remains beneath the ‘low in total sugar’ threshold (5 grams or less per 100 g of food) as defined by the UK nutrition and health claims guidance (60), it is difficult to determine the breakdown between naturally occurring versus added sugars in PBAs. This distinction is important because high intake of added sugars is strongly associated with adverse health outcomes (61–63). Therefore, consumers who replace meat and dairy with PBAs should keep in mind their sugar intake, especially from added sugars.

Understanding the micronutrient profile of PBAs is complex; not all studies report micronutrient content, and fortification is inconsistent within and across countries (64, 65). Consequently, concerns regarding potential micronutrient deficiencies are frequently raised in scientific debates comparing PBAs with animal-based foods.

While more clinical trials are needed to examine micronutrient absorption across all PBAs, current evidence on plant-based drinks is mixed. For example, a study conducted by Dineva et al. (52) found significantly lower iodine intake in exclusive consumers of almond and soy drinks. In our review (33), we found that when PBAs are fortified, they generally match their animal-based counterparts in contributing to the delivery of the recommended daily allowance of key micronutrients like iron, calcium and vitamins B12, B2 and D. A modelling study also demonstrated that incorporating optimised plant-based meats (fortified with iron and zinc), resulted in nutrient-adequate and overall healthier diets than current baseline diets (66). While fortification may allow PBAs to match their animal-based counterparts in delivering key micronutrients, fortification is not a standard practice. In our study we observed that from 1259 PBA products with listed nutritional profiles, only 502 (40%) reported micronutrients (33), suggesting that many were probably not fortified. These large micronutrient variability in PBAs may pose significant risks, especially for vulnerable populations like children. This is because observational evidence suggests that children consuming unfortified plant-based drinks have lower BMI, height, and serum vitamin D concentrations compared with those who consume cow’s milk (67).

Furthermore, studies reporting micronutrient composition usually rely on a limited number of micronutrients from the front-of-pack food labels rather than using analytical samples. A recent study that collected data from national food composition tables found that, regardless of fortification, plant-based meats can be a source of several types of micronutrients such as alpha-linolenic acid, folate, vitamin E, vitamin K, calcium, magnesium, manganese, copper and iron (68).

This evidence suggests that if carefully selected plant-based meat analogues can adequately contribute towards a healthy diet, particularly when used as partial replacements. However, until micronutrient evidence improves through more comprehensive analytical studies, careful consideration is needed when contemplating complete dietary replacements of animal-based foods with PBAs (69). This caution is especially warranted given that modelling studies indicate complete replacement of animal-based foods with PBAs could increase micronutrient deficiency risks of iodine and vitamin B12 (for females) (70), zinc (for males) (70) and n-3 long-chain fatty acids, vitamin B12, calcium, iron, iodine and riboflavin in the general adult population (70–73). These concerns become even more prominent among vulnerable groups such as children, pregnant women, the elderly, and those with chronic illnesses, as these subgroups have distinct nutritional needs than the general population.

### Environmental impacts

In the following section, the evidence on plant-based meats and dairy is considered in relation to their environmental impacts. There is a growing number of studies evaluating the environmental impacts of plant-based meats and drinks, focusing primarily on mycoprotein-, soy- or pea-based meats and almond- and soy-based drinks (33). However, data is missing for other plant-based drinks, and other product types such as plant-based yoghurt, cheese or egg analogues. The environmental impacts in these studies are usually measured using the life cycle assessment methodology, but studies usually focus on greenhouse gas emission, land use and water use metrics (33).

Partial or complete substitution of animal-based products with similar PBAs have a median reduction ranging from -53% to -94% for greenhouse gas emissions, -57% to -90% for land use and -93% to 4601% for water use (33). They are, therefore, a valuable strategy to help individuals shift to more sustainable diets and contribute to net-zero targets. However, differences in methodologies, study context and data choices that influence environmental outcomes should always be handled with great care. While reductions are reported consistently across several studies (29–32, 74, 75), Nájera Espinosa et al. (33) found a few data points where PBAs had a higher footprint and some extreme outliers, particularly for water use, for single studies on almond-based drinks (76) and soy-based meat (77). These outliers and single data points may introduce uncertainty, complicating policy recommendations and potentially slowing action to change. It is essential to carefully examine these extreme cases, assess their validity, and include multiple data points to ensure reliable environmental recommendations.

While PBAs offer environmental benefits, the identified benefits on greenhouse gas emissions, water, and land use provide only a partial view of the environmental impact of PBAs. Other crucial metrics—such as energy consumption, water and soil pollution, and biodiversity loss—are less commonly studied. The limited evidence may be due to lack of funding and data sources for other environmental impact categories. The reliance on a limited number of crops (such as soy, pea, wheat, oats, mycoprotein, and coconut oil) could lead to other long-term environmental, social, food security, and economic challenges. For example, heavy dependence on monoculture not only harms biodiversity but also degrades soil, increases pests and diseases, and concentrates production within an industrial agri-food system reliant on global supply chains and trade dependencies (78–80). This dependency poses food security risks and makes prices vulnerable to fluctuations within industrial monoculture, which could drive further reformulation of PBAs if certain ingredients become scarce. While the evidence is consistent and the direction of the environmental benefits is evident that can guide policy and practice, further research should continue to explore potential trade-offs that may not yet be fully understood.

## Processing of plant-based analogues

In this section, the evidence on plant-based meats and dairy is considered in relation to their processing level. Although processing offers benefits such as improved food safety, extended shelf-life and facilitates fortification (81), accumulating evidence associates consumption of ultra-processed foods with negative health effects (82–85). Based on the level of processing and use of ingredients such as food additives, most PBAs technically fall in the ultra-processed foods category according to the NOVA classification (86).

However, it is critical to recognise that not all ultra-processed foods are nutritionally equal, especially since this categorisation does not account for nutritional outcomes. While NOVA’s main goal is to guide consumers away from replacing wholefoods with ultra-processed foods, categorising PBAs within this classification system overlooks the fact that PBA products, when used as replacements for less healthy options such as processed meats and sweetened dairy products, can offer better nutritional outcomes whilst also offering major environmental benefits. A nutritional distinction is essential because the nutrient profiles of PBAs are usually differ considerably from those found in ultra-processed foods, although there are some exceptions (86). Furthermore, a recent study proposing a more nuanced subclassification of various ultra-processed food groups found that plant-based meat analogues, in particular, were not associated with health risks like those linked to ultra-processed foods such as cakes, biscuits, confectionary and alcohol (87).

The nuanced categorisation, proposed by Cordova and colleagues’(87), suggest that further disaggregation of PBAs is needed for accurate interpretations of the health benefits. Many studies often compare broad categories of PBAs with their animal-based counterparts (i.e. plant-based drinks with dairy milk or plant-based meat with meat & poultry) or generalise the findings of specific PBAs to the entire category. For example, a recent meta-analysis suggested that plant-based meats can modestly improve cholesterol and slightly reduce weight, however, mycoprotein-based meats had greater positive effects in comparison to other plant-based meats (28). Similarly, a systematic review comparing soy drinks with cow’s milk identified positive cardiometabolic effects (55), yet these findings cannot be generalised to other types of plant-based drinks.

## Future directions

Taking our findings into consideration, several key areas emerge as essential for advancing the field to avoid unintended consequences. In the following sections, we propose key research directions organised by theme to better inform policy and practice.

### Strengthen the nutritional evidence base

Nutrient data from labels and web-scraping do not provide a complete assessment of nutritional composition of PBAs, and the assumption that certain micronutrients are absent because they are not reported on labels is not entirely accurate. National food composition tables include analytical samples reporting additional micronutrients that are not usually mentioned on food labels (e.g. magnesium, phosphorus, tryptophan) (88). Global food composition tables range from limited PBA entries (e.g. UK) to more complete nutrient profiles (e.g. Netherlands) for various disaggregated categories of PBAs such as plant-based meats by type of product (e.g. nuggets, mince, sausages, meatballs, burgers) and main ingredient (e.g. soy, pea, wheat), as well as plant-based drinks (e.g. soy, almond, oat, coconut), plant-based yogurts (e.g. soy, coconut) by main ingredient, and similarly for other products such as plant-based cheese and ice-cream (89). Future studies should align data sources and avoid comparisons of PBA label data with analytical samples for animal-based products.

### Better comparisons between PBA categories for dietary recommendations

To guide food-based dietary recommendations, broad nutrition comparisons of PBA are insufficient for identifying healthier options. To navigate the complex nutritional variability, careful considerations are required. Research should be grounded in like-for-like comparisons (i.e. mincemeat vs plant-based mince or bacon vs plant-based bacon) and sub-classification by primary ingredient (e.g. soy, almond, oat, pea). This approach would distinguish nutrient-dense PBAs from typical unhealthy PBAs. Thus, providing clearer guidance for consumers and policy recommendations, and facilitating further health research, given likely differences in biological responses across subcategories.

### Guidance for consumers and vulnerable groups

Evidence on micronutrient bioavailability and bioaccessibility in PBAs is limited, with some studies suggesting lower bioavailability for certain micronutrients (31). Further research is there required, as lack of evidence may pose risks to consumers of PBAs in both the general population and vulnerable groups (90, 91). In addition, PBA manufacturers could help address these challenges by improving nutritional value, bioavailability and bioaccessibility through various pathways. For example, exploring innovative processing techniques to improve nutrient uptake by using strategic ingredient combinations (e.g. grains and legumes, vitamin C with iron fortification), increasing the proportion of wholefoods (vegetables, legumes), or reducing refined ingredients, sodium and added dailys (92, 93). These combinations could lead to enhanced protein and fibre content and iron bioavailability of PBAs, hence improving their overall nutritional quality.

Clearer labelling and improved nutritional food standards would support consumer’s food choices. National dietary recommendations may require tailored guidance for vulnerable groups, considering their dietary patterns and the specific animal-based foods being replaced. Because consumers generally purchase PBAs along with animal-based foods (34, 94), both short- and long-term health studies could broaden the “plants versus animals” evidence by evaluating the role of selected PBAs as part of a balanced, health-promoting diet that also includes plant-based wholefoods (e.g. legumes, vegetables and nuts) and some animal-based foods.

At the macronutrient level, PBA protein content is typically not higher than in their animal-based counterparts, though many products, especially plant-based meats, still qualify as a “source of” or “high in” protein according to the UK nutrition and health claims standards (60). While protein intake remains a topic of debate in the scientific community, protein sufficiency is less of a concern for many high-income countries since protein deficiency has minimal impact on overall burden of disease (58). In contrast, protein overconsumption, particularly from red and processed meats, is more prevalent and linked to negative health outcomes (3, 49, 95).

Nonetheless, recommendations in relation to PBAs should be made for sub-groups with higher protein needs (e.g. children, elderly) (96). For example, mycoprotein-based meats and soy-based drinks/yoghurt can provide optimal or complete amino acid profiles (97–99). While some studies suggest that PBAs have lower amino acid levels compared to their animal-based counterparts (with exceptions) (98, 100), others indicate that plant-based proteins can complement animal-based foods (101). There is an opportunity to improve nutrient absorption by optimising ingredient combinations at the manufacturing level, which could both increase food diversity and enhance absorption and intake of essential amino acids from plant-based sources to support health (102). Simultaneously, education campaigns should highlight the complementary profiles of PBAs to encourage partial replacements (101), with careful attention to vulnerable groups with special dietary requirements.

### Processing, additives and health

Future research on both ultra-processed foods and PBAs is needed to fully comprehend the role of: high palatability of PBAs, satiating effects, changes in the food matrix, nutritional profiles, isolated ingredients (such as protein isolates and hydrolysed proteins), and by-products formed during packaging and processing. All of which may influence endocrine pathways and gastrointestinal health (87).

More research is also needed to understand the gastrointestinal impact of the quantities and types of different ingredients used in PBAs. While food additives commonly used in products undergo safety assessments based on country- or region- consumption data, the health effects of consuming multiple additives together remain unclear. Further research is needed to explore the impacts of regular consumption of food additives and potential “cocktail” effects, through PBAs and ultra-processed foods. This includes investigating how additives may contribute to dysbiosis and its potential effects on brain function and behaviour (103).

### Standardised environmental outcomes

Globally, numerous databases report on environmental footprints (e.g. Agribalyse, Ecoinvent, World Food LCA database, OpenLCA Nexus), yet the assessment of food-related footprints is still novel in the nutrition field and especially for PBAs. Standardised environmental methods are essential to ensure comparability and prevent misinterpretation. Establishing a national guideline—like an environmental food national guideline or adding footprints to food composition tables—could improve data selection and transparency. Such a guideline would strengthen the evidence base and support interventions, such as environmental footprint labelling (e.g. carbon and water footprint labelling) to help consumers choose lower-impact food options like PBAs and traditional plant-based foods (i.e. tofu and tempeh) (104, 105).

### Beyond single ingredients: innovation and food systems effects

As climate change continues to affect crop yields and quality (106–109), diversifying PBA ingredients could reduce dependence on a narrow set of crops, enhance nutritional diversity, and support food system resilience. Rapid technological innovation can support such shifts by introducing new inputs [e.g. fungi-based Fy Protein™ (USA market) (110), animal-free dairy protein made by microflora (in USA market) (111), and potato and avocado drinks (in UK/USA markets)]. Nonetheless, the environmental impacts of ingredient substitutions should be carefully evaluated as demand scales. For example, in 2024, a USA-based leading manufacturer of plant-based meat analogues (Beyond Meat), announced that its fourth-generation product would be formulated with avocado oil instead of palm oil to help reduce cholesterol (112). While this change may benefit health, it could increase water-related impacts relative to palm oil. Although several environmental impacts have been documented for palm oil, this oil is considered relatively efficient and more economically viable compared to other vegetable oils (113, 114). Avocado cultivation on the other hand, often requires significant water and is frequently grown in water-stressed regions (115), with limited environmental data on large-scale avocado oil production. These impacts underscore the need for holistic assessments that consider both health and environmental effects of ingredient choices (115).

Beyond ingredients, recent work suggests there is an underestimation of the environmental footprints of dairy and meat in ready meals (116, 117). Incorporating PBAs (partially or completely) into ready meals could result in additional environmental benefits since 88% of the adult UK population consumes ready meals regularly (118), with the majority of these meals containing meat (70% until 2021) (119). Expanding research to evaluate health and environmental trade-offs in ready meals containing PBAs presents a valuable opportunity to encourage consistent shifts in dietary patterns.

## Policy relevance and practice

In this last section, we discuss the role of PBAs for policy relevance and practice. Efforts to increase the uptake of selected PBAs should not come at the expense of replacing foods that are culturally appropriate, traditional and known to be healthier and better for the environment. These include traditional plant-based foods (e.g. tempeh, tofu, falafel, nut roast, baked beans), plant-based wholefoods (e.g. legumes, nuts and seeds) and dishes that replace meat with legumes or vegetables (e.g. vegetable chilli) (120). Prioritising the promotion of these plant-based foods should remain a key focus, however in contexts with high consumption of processed foods, such as in the UK, integrating PBAs could provide a transitional step towards a more plant-forward diets (121).

Given the large potential of dietary changes to address environmental challenges and the positive health and environmental outcomes linked to the consumption of PBAs, greater efforts are needed to promote the consumption of both healthy and sustainable PBAs along with plant-based wholefoods. However, various gaps must be addressed to inform public health and environmental policies better. One potential restraint is the limited funding available for research on PBAs, largely due to their relative novelty. Many studies are funded by PBA manufacturers, which raises concerns about bias, though outcomes appeared consistent regardless of funding source (33). Nonetheless, it remains essential for independent institutions to invest in PBA research or institutions such as the UK National Alternative Protein Innovation centre (122) to rigorously assess both the benefits and challenges of these products. Future assessments should also integrate food safety into research. Food safety was often overlooked in nutritional, health and environmental studies, despite identified chemical, biological and physical food safety threats (123, 124).

The wide nutritional variation in PBAs impacts their reliability as direct replacements for animal-based foods in several ways, especially regarding the micronutrient content, protein quality, fortification consistency and ingredients. This lack of standardisation may discourage consumption and pose risks for exclusive or non-exclusive consumers of PBAs and vulnerable groups with specific dietary needs. To mitigate health risks and help consumers navigate the varying nutrient profiles of PBAs, simplifying food labels and clearly communicating the origin of raw ingredients is needed. For example, food labels could indicate fortification levels or highlight the potential benefits and risks of replacing comparable animal-based foods. Current recommendations in the UK food-based dietary guidelines suggest consumption of fortified soy-based drinks and mycoprotein-based meat (51). These recommendations could be extended to encompass a wider selection of PBAs, to increase uptake of those PBAs that can contribute to a healthy diet. A careful selection of PBAs in food-based dietary guidelines could support the promotion of adequate PBAs for public procurement in institutional catering (e.g. schools, hospital, universities), especially as alternatives to replace processed meats and to accommodate dairy milk allergies.

Even though there are some environmental differences between various types of PBAs made with different ingredients, most PBAs demonstrate substantial environmental benefits compared to animal-based foods, particularly red and processed meat.

A larger range of recommended PBAs would not only improve social acceptance by offering consumers more informed choices and variety but also support food procurers and chefs in accommodating allergies and preferences with different PBAs. While some consumers of PBAs have transitioned towards these products due to lactose intolerance or milk protein allergy, new sources of plant-based proteins like pea protein or mycoprotein may also pose risks to certain individuals with allergic sensitivities (123). A larger range of healthier PBAs would help accommodate these personal barriers while also introducing different types of PBAs, increasing familiarity and potentially encouraging greater adoption at home.

To avoid unintended consequences, the application of nutritional standards for PBAs would help reduce potential nutritional related risks. In high-income countries like the UK, there are well established compositional standards for various products including meat and dairy products (125). For example, pork sausages must contain 42% of pork before being called ‘pork sausages’ (126, 127), lean mincemeat cannot contain more than 7% of fat (128), and full fat milk must contain at least or equal to 3% fat (129). Research in the field shows there is manufacturing capacity to create healthier products within the wide nutritional ranges of PBAs with products with more than 40% of whole ingredients like legumes and vegetables (33), or “health-boosting” plant-based meat enriched with amino acids like lysine (40).

Considerations of the development of regulations or standards for PBAs could enable a larger range of healthier PBAs and less confusing messaging to consumers. For example, minimum fortification standards for PBAs with key micronutrients could deliver on key nutrients that are under-consumed in many high-income countries (130), supporting wider national targets for the reduction of nutritional deficiencies. It could also support individuals following diets that do not contain animal-based products (e.g. vegans), and other population sub-groups such as pregnant women, children and the elderly who may have “higher than average” micronutrient needs. Limiting nutrients of concern in PBAs formulations such as saturated fat, added sugars and sodium is another alternative that could provide large benefits to high-income population’s health overall.

From the regulatory angle, there have been great efforts to reduce free sugars. Moving forward it is important to guide manufacturers against substituting free sugars for non-sugar sweeteners. Emerging evidence suggests potential health risks associated with non-sugar sweeteners, while the food safety of other additives is unclear(131, 132). Regulations of this type would also allow consumers to choose PBAs they like, rather than having to choose between products based on the presence or absence of certain nutrients. The implementation of a food safety surveillance system for PBAs, alongside consumer education on proper handling and storage practices (e.g. always refrigerate plant-based milks) is also recommended. While surveillance systems usually record and monitor food safety-related activities, such systems could also be implemented to monitor and keep track of the fast pace of the industry and any nutritional variations in PBAs to help stakeholders stay up to date.

Additionally, food manufacturers of PBAs have communicated the importance of producing PBAs with healthier nutrient profiles than animal-based foods (81). This is because PBAs are designed to directly replace animal-based products, which have traditionally been sources of key nutrients in diets. Although nutritional composition may not be a primary driver of consumer choice or manufacturer decision-making in the short-term, large nutritional discrepancies have the potential to influence public health outcomes and may, over time, affect product acceptance and market sustainability. By hiring external consulting companies to assess the environmental footprints of PBAs exemplifies manufacturers’ attempt to ensure reliability. For this reason, manufacturers of PBAs have focused primarily on matching the nutritional profiles of animal-based foods. Likewise, researchers have tended to compare PBAs mainly with the nutrient content found in animal-based foods. While fortification of PBAs with micronutrients commonly found in animal-based foods (e.g. iron, iodine, calcium, vitamin B12) should remain a priority, focusing solely on these micronutrients may represent a missed opportunity. PBAs could also be fortified with additional nutrients to broaden their nutritional contribution beyond that of animal-sourced products, by providing nutrients that are not easily obtained from animal-based foods yet are also lacking in the general population (e.g. fibre, vitamin D) (133, 134). Research on other compounds not necessarily present in animal-based foods such as chitin and β-glucan found in mycoprotein-based meats (135), or health-promoting substances such as water-soluble bioactive compounds present in plant-based beverages (e.g. flavonoids, phenolic acids, vitamins, carotenoids, and other phenolics) (136), could be beneficial when assessing nutritional and health outcomes.

Another overlooked opportunity in PBA production is the use of nutrient-rich crops and under-utilised crops, alongside a higher proportion of commonly consumed wholefoods, to enhance the fibre content and overall nutritional value, while reducing refined ingredients. Including under-utilised crops could provide a win-win scenario for nutrition and the environment. Under-utilised crops can further enhance the nutritional content of PBAs, reduce climate change impacts and improve biodiversity (137). Relying on under-utilised crops may also create opportunities to improve farmers livelihoods by integrating them into the value chain if they choose to reduce their livestock production.

Further research is also required to understand who is willing to consume PBAs and why, and how to effectively promote PBAs that are both healthy and environmentally friendly. Supportive regulatory measures and further efforts from the food industry, could improve the affordability, availability, and sensory appeal of PBAs (138). However, understanding consumer behaviour is essential for considerations of certain measures (e.g. fiscal measures) given that emerging studies suggest that consumers would expect much lower prices for PBAs to swap from animal-based foods (139–141). It is equally important to inform both consumers and non-consumers of PBAs about processing methods, and the health, nutritional and environmental advantages and challenges of incorporating PBAs—whether partially or fully—into their diets. Such efforts should highlight the importance of not replacing plant-based wholefoods with PBAs.

From the manufacturing perspective, PBAs are targeted to the average consumer. However, evidence shows the importance of taste, texture and cost for a higher uptake of plant-forward diets (8, 142, 143). PBA manufacturers could make further improvements to enhance product appeal and reduce costs (144–146). Additionally, the emerging technologies used to mimic the sensory experience of meat and dairy presents an opportunity to incorporate beneficial dietary compounds, supporting groups who may have special dietary needs (e.g. children, adults with underlying health conditions).

The regulatory side could support this field through carbon taxes on animal-based foods, reallocating subsidies away from livestock production, or providing support schemes for farmers to transition from livestock production to plant crops (147). The implementation of tailored subsidies for plant-based foods (139), including healthier PBAs and other alternatives to animal-based products (e.g. plant-based whole foods, traditional plant-based alternatives), could help alleviate the economic burden for some individuals. Recent studies have suggested fiscal measures could accelerate positive shifts, benefiting individual’s health, the environmental and social well-being (148, 149). For example, lowering prices by 20-40% has shown increased purchases of healthier food choices [(150) & Xu et al. in (9)].

## Conclusion

In conclusion, to achieve healthy and environmentally sustainable diets, plant-based wholefoods should remain the primary objective. However, given the limited adoption of plant-based wholefood consumption, PBAs represent a viable approach when seeking to reshape food systems by acting as a transitional bridge for consumers. When carefully selected, PBAs often demonstrate good health and environmental outcomes compared to their animal-based counterparts, particularly when substituting for frequently consumed processed meats. Nevertheless, the considerable nutritional variability among PBAs and the lack of comprehensive long-term health studies underscore the necessity for further research. Such research is critical for developing evidence-based policies and practices, and for expanding the range of healthier PBAs.

## Figures and Tables

**Table 1:** Summary of key findings on the health, nutritional and environmental outcomes of plant-based analogues (PBAs).

PB category	Health outcomes	Average nutritional content* comparison (vs. ABFs**)	Average environmental impact (vs. ABFs**)
**Meats**	**Types were evidence is available:** mycoprotein & legume-based **Pros:** Improved glycaemic markers, insulin, and cholesterol; lower energy intake and cardiovascular risk; positive microbiome changes **Focus:** Short-term studies (<1d-56d) on healthy/overweight adults **Considerations**: Outcomes may vary by population	**Types were evidence is available:** Mycoprotein, nuts & seeds, cereals, fruits & vegetables, & legume-based **Pros:** ↑ Fibre; ↓ Saturated fat & energy density **Cons:** Risk of Iron & vit B12 deficiency if not fortified; ↑ total sugar; some have ↑ sodium; not all match ABFs protein content **Healthiest:** Mycoprotein & legume-based	**Types were evidence is available:** Mycoprotein, nuts & seeds, cereal, fruits & vegetables, & legume-based **GHG:** -90% **Land:** -85% **Water:** -77%
**Drinks**	**Types were evidence is available:** almond & soy-based **Pros:** Improved blood lipids and blood sugar (+ cardiovascular health); similar glycaemic response to dairy milk when consumed with carbohydrates **Cons:** Micronutrient deficiency risks exclusive consumers (Iodine/vit B12); dental health for sweetened types **Focus:** Short-term studies (<1d-30d) on healthy adults	**Types were evidence is available:** Coconut, nuts & seeds, cereals, fruits & vegetables & legumes-based **Pros:** ↑ Fibre; ↓ Saturated fat & energy density **Cons:** Risk of calcium & iodine deficiency if not fortified; protein often lower than dairy (except legume-based); ↑ total sugar **Healthiest:** legume-based	**Types were evidence is available:** Nuts & seeds, cereals, fruits & vegetables & legumes-based **GHG:** -84% **Land:** -72% **Water:** -62%
**Cheese**	**Types were evidence is available:** Limited Mixed metabolic effects; grouped with other PBAs products	**Types were evidence is available:** Limited; mainly coconut-oil-based **Pros:** ↓ total sugar & sodium **Cons:** Often ↑ energy density and saturated fat; many lack fibre	**Types were evidence is available:** Limited; mainly coconut-oil-based **GHG:** -75% **Land:** -83% **Water:** -45%
**Yoghurt**	**Types were evidence:** Limited Mixed metabolic effects; grouped with other PBAs products	**Evidence:** Limited; no disaggregated data **Pros:** ↓ Saturated fat & sodium **Neutral:** Similar energy density to dairy versions	**Types were evidence:** Limited; No land/water data **GHG:** -59%

* Most nutritional data is derived from web-scraping or product labelling

** Comparison with median values

ABFs: animal-based foods; PB: plant-based; PBAs: plant-based analogues; d: day; vit: vitamin; GHG: Greenhouse gas emissions; Land: Land use; Water: Blue water footprint; +: positive
